# Neuropsychiatric complications 3–4 years after stroke: a population-based study of fatigue, depression and cognition

**DOI:** 10.1136/bmjopen-2024-096908

**Published:** 2025-07-06

**Authors:** J Aked, Hossein Delavaran, Fredrik Wennerström, Arne Lindgren

**Affiliations:** 1 Department of Clinical Sciences Lund Neurology, Lund University, Lund, Sweden; 2 Department of Medicine, Blekinge Hospital, Karlskrona; 3 Department of Cardiology, Danderyd University Hospital, Stockholm, Sweden; 4 Department of Emergency Medicine, Skåne University Hospital, Malmo, Sweden; 5 Department of Neurology, Rehabiliation Medicine, Cognitive Disorders, Geriatrics, Skåne University Hospital Lund, Lund, Skåne, Sweden

**Keywords:** Stroke, Cognition, Fatigue, Depression & mood disorders

## Abstract

**Abstract:**

**Objectives:**

To study the prevalence of and interplay between common neuropsychiatric sequelae 3–4 years after onset of first-ever stroke—specifically post-stroke fatigue (PSF), post-stroke depression (PSD) and post-stroke cognitive impairment (PSCI).

**Design:**

Population-based cohort study.

**Setting:**

Catchment area of a Swedish University Hospital.

**Participants:**

We recruited individuals with first-ever ischaemic stroke or intracerebral haemorrhage in the initial cohort; 151 of these died prior to follow-up and 47 (12%) were lost to detailed follow-up. We followed up 202 individuals with median age: 72 (IQR 65–79), 40% female, either in clinic, via home visits or via telephone.

**Primary and secondary outcome measures:**

Primary outcome measures included PSF (Fatigue Assessment Scale), PSD (Patient Health Questionnaire-9) and PSCI (Montreal Cognitive Assessment). Secondary outcome measures included dependency in activities of daily living (ADL; Barthel Index), health-related quality of life (HRQoL; Short-Form Questionnaire-36, EuroQoL-5D and Stroke Impact Scale) and stroke severity (National Institutes of Health Stroke Scale (NIHSS)).

**Results:**

Significant PSF was present in 46/195 (24%), PSD in 21/191 (11%), and PSCI in 93/173 (54%) respondents. Among 169 participants with available data for all three domains, 100 (59%) had impairment in at least one domain. Participants with PSCI were older than those without (median: 75 vs 67 years; p<0.01), but age did not differ for those with/without PSF or PSD. Among 21 respondents with PSD, 20 (95%) had PSF. PSD and PSF were strongly correlated (ϱ=0.69; p<0.01) and PSF was associated with worse HRQoL (HR: 1.15; 95% CI 1.08 to 1.22; p<0.001). ADL dependency was associated with worse NIHSS at follow-up (B: −3.81; 95% CI −4.77 to −2.85), baseline home care (B: −18.34; 95% CI −26.95 to −9.73) and PSF (B: −0.65; 95% CI −1.05 to −0.26).

**Conclusions:**

PSF, PSD and PSCI are highly prevalent 3–4 years after stroke. PSF and PSD overlap and correlate. PSF is associated with ADL dependency and worse HRQoL. Clinical awareness and research of treatment for neuropsychiatric complications of stroke are needed.

**STRENGTHS AND LIMITATIONS OF THIS STUDY**

- The main strength of this study is its comprehensive population-based cohort, assuring minimal selection bias.
- This study also assesses several neuropsychiatric sequelae concomitantly, in the medium-long-term chronic phase of stroke, thereby excluding transient fatigue or cognitive deficits in the acute phase.
- Assessments used in the present study are commonly used in stroke rehabilitation research which allows for direct comparison with other populations.
- The study is limited by a non-negligible (12%) proportion of individuals lost to detailed follow-up.
- The study does not include a control population to allow comparison of cognitive impairment with age-matched individuals without stroke, and data on the participants’ pre-stroke function are also lacking.

## Introduction

 Stroke is the second most common cause of death, a leading cause of acquired disability worldwide, and the global burden of stroke is projected to increase in coming decades.^1 2^ Various functional impairments can follow a stroke and affect body functions, activities and participation of stroke survivors long term.^3^,^7^

Alongside neurological sequelae such as aphasia, paresis, ataxia and visual impairments with impact on independency in daily living, other common long-term consequences of stroke include neuropsychiatric conditions such as fatigue,^6^ depression and anxiety,^8^ and cognitive impairments.^9 10^

Post-stroke fatigue (PSF) has an estimated prevalence of between 25 and 85% among stroke survivors and is reported by 40% of patients as one of their worst symptoms.^6^ PSF has been associated with depression^11^ and worse function in complex activities of daily living (ADL).^12^ Post-stroke depression (PSD) and other affective disorders are common after stroke, affecting approximately one-third of stroke survivors at some time post-stroke, with a prevalence that is significantly higher than among the background population.^13 14^ Post-stroke cognitive impairment (PSCI) occurs in 7–41% of stroke survivors within 1 year after stroke onset,^13^ and in a Swedish cohort—46–61% had cognitive impairment at 10 years after stroke.^14^ PSCI is associated with limitations of activity, including basic and complex ADL.^15^

The wide spans of prevalence of PSF, PSD and PSCI may in part be caused by different definitions of these conditions. Moreover, there is a lack of recent and especially population-based studies on long-term outcome after stroke across these multiple domains of functioning. Multimodal long-term outcome data are important for evidence-based planning of healthcare resources, for prioritising other research efforts and for aiding rehabilitation.

In this population-based study of first-ever stroke patients at 3–4 years after stroke, we aimed to assess (1) long-term prevalence of neuropsychiatric and cognitive complications, (2) predictors of neuropsychiatric complications, and (3) relationships between different neuropsychiatric complications of stroke.

## Methods

### Population-based cohort

The population-based cohort in the present study has previously been described in detail.^16^ Briefly, we recruited adult individuals with first-ever stroke according to the WHO definition^17^ between 1 March 2015 and 29 February 2016, in the local catchment area of Skåne University Hospital in Lund, Sweden. Recruitment was both prospective and retrospective from multiple overlapping sources, in agreement with proposed gold standard criteria for epidemiological stroke studies.^18^ Individuals with ischaemic stroke (IS) or intracerebral haemorrhage were included, but not those with subarachnoid haemorrhage. Traumatic and iatrogenic stroke cases were also excluded.

### Baseline characteristics

The cohort’s baseline characteristics and their ascertainment have been previously described.^16 18 19^ Stroke symptom severity was assessed using the National Institutes of Health Stroke Scale (NIHSS) by certified research nurses in-hospital, or by review of medical records when necessary.^20^ Comorbidities at baseline were quantified with the Charlson Comorbidity Index (CCI).^21^ As in prior studies of this cohort, IS was stratified into the following: (1) clinical syndromes according to the Oxfordshire Community Stroke Project defined as follows: lacunar infarct, partial anterior circulation infarct, total anterior circulation infarct, and posterior circulation infarct;^22^ and (2) pathogenetic mechanisms of IS according to the Trial of ORG 10172 in acute stroke treatment: cardio-aortic embolism, large-artery atherosclerosis, small artery occlusion, other causes, and undetermined cause.^23^

### Follow-up procedure

We previously described the follow-up procedure in detail.^19^ In brief, we conducted follow-up visits with participants at 3–4 years after first-ever stroke via clinic, home or telephone visits and assessed the following: (1) functional status using modified Rankin Scale (mRS);^24^ (2) dependency in ADL with Barthel Index (BI);^25^ (3) health-related quality of life using Euro-QoL-5D;^26^ Short Form Survey, question 1 (SF-36);^27^ and Stroke Impact Scale V.2.0;^28^ and stroke severity at follow-up with NIHSS.^20^

### Assessments at follow-up

In addition to previously described assessments at 3–4-year follow-up,^19^ we also assessed the patients’ neuropsychiatric condition regarding (1) PSF with the Fatigue Assessment Scale (FAS),^29^ (2) depressive symptoms with Patient Health Questionnaire-9 (PHQ-9)^30^ and (3) PSCI with the Montreal Cognitive Assessment (MoCA).^31^ The Swedish V.7.0 of MoCA was used, with a range from 0 to 30 points, where 0 indicates the most severe cognitive impairment. An extra point was given to those with less than 12 years of formal education.^31^ The cut-off for significant cognitive impairment was ≤24 points on MoCA.^32^ MoCA results were further stratified according to severity as follows: no cognitive impairment (score 25–30), mild cognitive impairment (score 20–24), severe cognitive impairment (score 0–19). We used a Swedish translation of FAS to assess PSF with a range from 0 to 50 points where 50 points indicate most severe PSF. We used a cut-off of ≥24 points on FAS to signify significant PSF.^29^ The PHQ-9 ranges from 0 to 27 points, where 27 points indicate most severe depressive symptoms. A cut-off of ≥10 points was used to signify significant depressive symptoms.^30^ Both FAS and PHQ-9 were assessed via telephone interview when participants were ineligible for both clinic and home visits.

### Statistics

We assessed between-group differences using χ^2^ tests for categorical variables and Mann-Whitney U-tests for ordinal or non-normally distributed continuous variables. Spearman’s rho was used to assess correlations between outcomes at follow-up, and the Mantel-Haenszel test was used to determine if linear associations were present between outcomes.

We used binomial, ordinal logistic and multiple linear regression to determine predictors of neuropsychiatric outcome and relationships between functional and neuropsychiatric outcomes at 3–4-year follow-up using a model including the following: (1) a predictor model including age at baseline, sex, baseline stroke severity (NIHSS), living with or without care at baseline (as a proxy for pre-stroke function), stroke subtype, recurrent stroke between baseline and follow-up, and hypertension, diabetes mellitus, heart disease, hypercholesterolemia, and active smoking at baseline; and (2) a follow-up model including age at baseline, NIHSS at follow-up, CCI at baseline, living with or without care at baseline, living alone at follow-up, recurrent stroke, and PSF (FAS), depressive symptoms (PHQ-9), and cognitive impairment (MoCA) at follow-up. We tested regression models for multicollinearity and proportional odds, as well as independence of errors, similarity of variance across tested groups, outliers and normality for the linear models.

We used IBM SPSS Statistics V.28 for statistical analyses. P values<0.05 were considered significant although Bonferroni-adjusted p values are also presented where appropriate to adjust for multiple comparisons.

## Results

### Included patients and loss to follow-up

400 participants with first-ever stroke were included, among whom 151 (38%) died within 4 years before clinical follow-up. Baseline characteristics of the total cohort are shown in table 1. At a median time of 3.2 years (IQR: 3.1–3.5), 202 (51%) individuals were followed up in-person (n=180, 89%) or via telephone (n=22, 11%). Comparisons between those lost to detailed follow-up and followed up individuals have previously been reported.^19^

**Table 1:** Baseline characteristics among respondents to tests of neuropsychiatric outcomes

	Cognitive impairment,n=173			Fatiguen=195			Depressive symptomsn=191			Total cohortn=400
	PSCIn=93	No PSCIn=80	P value	PSFn=46	No PSFn=149	P value	PSDn=21	No PSDn=170	P value	–
Age, median (IQR)	75 (69–81)	67 (61–74)	<0.01	71 (66–79)	72 (64–79)	0.92	70 (61–77)	72 (65–79)	0.18	76 (68–84)
Sex, female, n (%)	41 (44)	24 (30)	0.06	21 (46)	54 (36)	0.30	8 (38)	65 (38)	1.00	180 (45)
Stroke subtype, n (%)			0.52			0.69			0.90	
IS	83 (89)	69 (86)		42 (91)	130 (87)		19 (90)	149 (88)		335 (84)
ICH	10 (11)	10 (13)		4 (9)	18 (12)		2 (10)	20 (12)		60 (15)
UND	0	1 (0)		0	1 (0)		0	1 (0)		5 (1)
NIHSS at baseline, median (IQR)	4 (2–9)	2 (1–4)	<0.01	4 (2–10)	3 (2–7)	0.11	5 (3–10)	3 (2–7)	0.05	5 (2–10)
Living situation at baseline, n (%)			0.01			0.76			0.54	
No home care	79 (85)	79 (99)		43 (94)	136 (92)		21 (100)	154 (91)		309 (77)
Home care or care facility	14 (15)	1 (1)		3 (6)	13 (9)		0 (0)	16 (9)		91 (23)
Baseline risk factors, n (%)										
Hypertension	75 (81)	51 (64)	0.02	41 (90)	103 (69)	<0.01	18 (86)	122 (72)	0.20	279 (70)
Diabetes mellitus	23 (25)	9 (12)	0.03	15 (33)	25 (17)	0.04	9 (43)	30 (18)	0.02	105 (26)
Heart disease	30 (32)	19 (24)	0.31	15 (33)	46 (31)	1.00	6 (30)	53 (31)	1.00	164 (41)
Smoking	14 (15)	12 (15)	1.00	8 (17)	24 (16)	0.82	5 (24)	27 (16)	0.36	67 (17)
OCSP (IS only), n (%)			0.03			0.10			0.28	
LACI	29 (35)	20 (29)		12 (29)	40 (31)		5 (26)	46 (31)		92 (28)
PACI	34 (41)	28 (41)		19 (45)	55 (42)		10 (53)	61 (41)		155 (46)
TACI	10 (12)	2 (3)		7 (17)	8 (6)		3 (16)	12 (8)		35 (10)
POCI	10 (12)	19 (28)		4 (10)	27 (21)		1 (5)	30 (20)		53 (16)

ICH, intracerebral haemorrhage; IS, ischaemic stroke; LACI, lacunar infarct; NIHSS, National Institutes of Health Stroke Scale; OCSP, Oxfordshire Community Stroke Project; PACI, partial anterior circulation infarct; POCI, posterior circulation infarct; PSCI, post-stroke cognitive impairment; PSD, post-stroke depression; PSF, post-stroke fatigue; TACI, total anterior circulation infarct; UND, undetermined.

### Missing data

There were ≥5% missing values for MoCA, PHQ-9 and NIHSS at follow-up (14%, 5% and 11%, respectively). These missing values were considered missing not at random since they were absent due to these participants’ inability to attend at in-person visits.

### Prevalence of neuropsychiatric outcomes

Significant PSF was reported by 46/195 (24%) respondents. The most common symptoms (reported as “sometimes to always present”) were “I am bothered by fatigue” (126/195; 65%), and “I get tired very quickly” (107/195; 55%). 16 (35%) of those with PSF reported that they always or often “did not have enough energy for daily life” (FAS, question 4).

The 3–4-year prevalence of significant post-stroke depressive symptoms was 21/191 (11%). The most common depressive symptoms (reported as present at least several days in the last 2 weeks) were “feeling tired or having little energy” (91/191; 48%), and “trouble falling or staying asleep, or sleeping too much” (75/191; 39%).

The 3–4-year prevalence of cognitive impairment was 54% among 173 respondents. Among the 93 respondents with cognitive impairment, the cognitive impairment was mild in 55 (59%) of cases. The most common impaired functions among the MoCA subtests were delayed recall (151/173; 87%), and visuospatial/executive functions (122/173; 70%).

In all, 100 of 169 (59%) participants who responded to all three outcome measures had a significant impairment in at least one of the domains. Baseline characteristics of respondents to the three outcome measures are shown in table 1.

### Predictors of neuropsychiatric outcomes

Age (OR: 1.08; 95% CI 1.04 to 1.12) and baseline stroke severity (NIHSS) (OR: 1.13; 95% CI 1.03 to 1.23) were significant risk factors for the presence of cognitive impairment (MoCA <25) at follow-up. In our predictor model, there were no significant independent predictors of PSF or depressive symptoms. Regression models and results are presented in online supplemental tables 1–3.

### Relationship between neuropsychiatric outcomes

In all, 100 of 169 (59%) participants who responded to all three outcome measures had a significant impairment in at least one of the domains. Participants with cognitive impairment were older than those without (median: 75 vs 67 years; p<0.01), while age did not differ across groups regarding PSF (median: 71 vs 72; p=0.92) or depression (median: 70 vs 72; p=0.18).

13 participants (8%) had significant cognitive impairment, PSF and depression concomitantly. Among those with PSF (n=46), 20 (43%) also had significant PSD. Meanwhile, 20 of the 21 (95%) individuals with significant PSD had significant PSF.

PSF and PSCI (ϱ=−0.13; p=0.06) and depression and PSCI (ϱ=−0.07; p=0.38) showed no or negligible correlation. However, PSF and depression were strongly positively correlated (ϱ=0.69; p<0.01).

**Figure 1:**
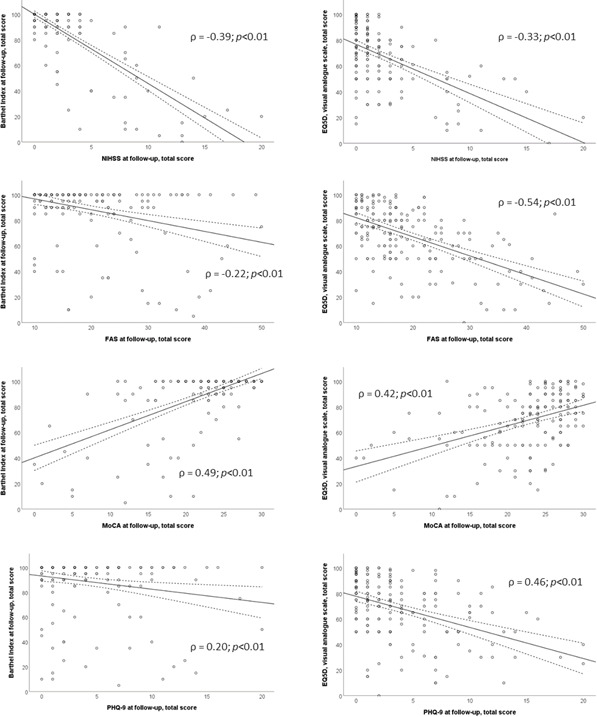
Scatter plots showing correlation between assessments at 3–4-year follow-up and functional outcome and HRQoL. EQ5D, Euro-QoL-5D (scale 0–100; 100=best outcome); FAS, Fatigue Assessment Scale (fatigue; scale 0–50; 0=best outcome); HRQoL, health-related quality of life; MoCA, Montreal Cognitive Assessment (cognition; scale 0–30; 30=best outcome); NIHSS, National Institutes of Health Stroke Scale (scale 0–42); PHQ-9, Patient Health Questionnaire (depression; scale 0–27; 0=best outcome).

**Figure 2:**
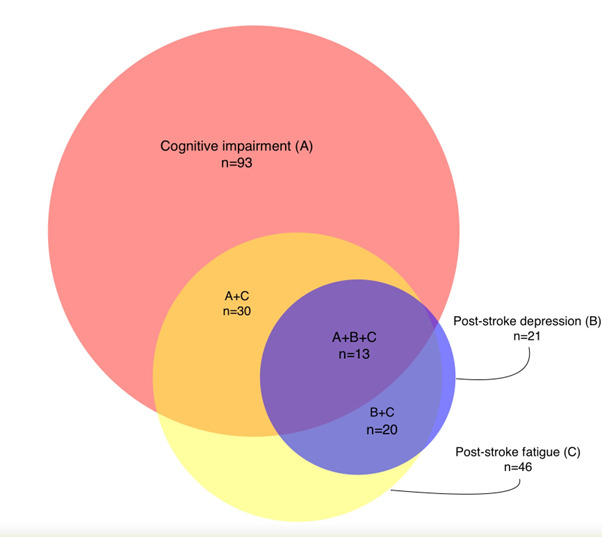
Proportional Euler diagram of the overlap between neuropsychiatric outcomes among the 202 stroke survivors. Post-stroke fatigue: ≥24 points, Fatigue Assessment Scale; post-stroke depression: ≥10 points, Patient Health Questionnaire-9; cognitive impairment: ≤24 points, Montreal Cognitive Assessment. Total number of respondents: post-stroke fatigue: n=195; post-stroke depression: n=191; cognitive impairment: n=173.

**Figure 3:**
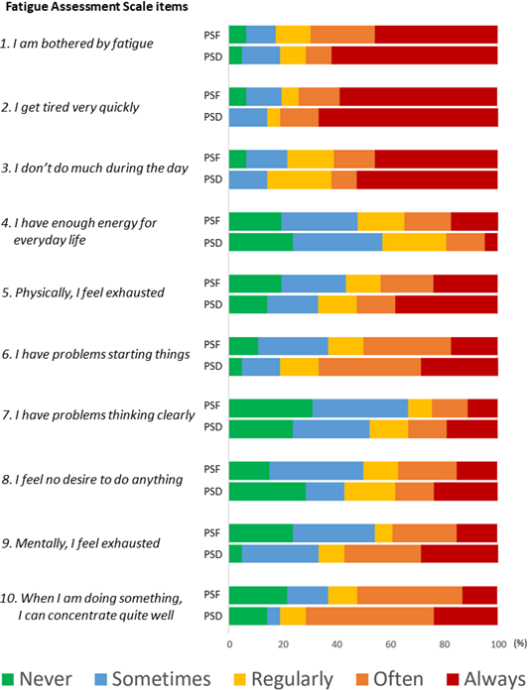
Response proportions to Fatigue Assessment Scale among participants with PSF and PSD. PSF indicates responses among those with significant PSF (n=46); PSD indicates responses among those with significant PSD (n=21). PSD, post-stroke depression; PSF, post-stroke fatigue.

Correlations between PSF, depression and PSCI are presented in figure 1, and their overlap is presented in figure 2. Responses to FAS and PHQ-9 among all individuals with PSF and all individuals with PSD are presented in figures3  4.

**Figure 4:**
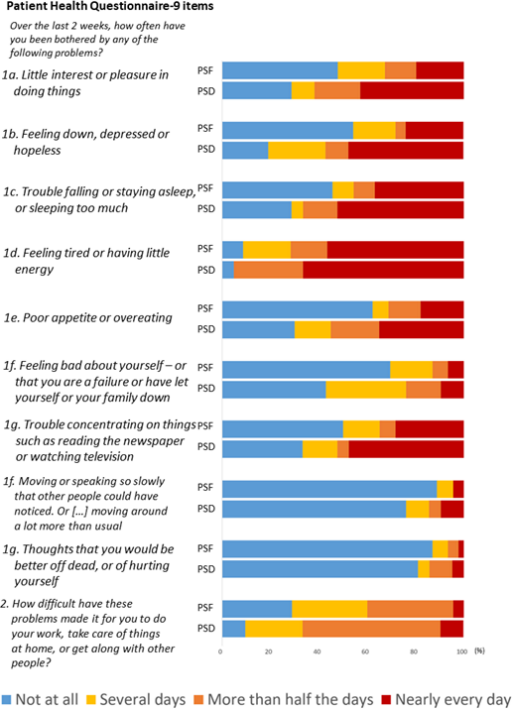
Response proportions to Patient Health Questionnaire-9 among participants with PSF and PSD. PSF indicates responses among those with significant PSF (n=46); PSD indicates responses among those with significant PSD (n=21). The colour legend for question 2 represents, in ascending order: not difficult at all, somewhat difficult, very difficult, extremely difficult. PSD, post-stroke depression; PSF, post-stroke fatigue.

### Associations between functional and neuropsychiatric outcomes

At follow-up, stroke survivors with poor functional outcome (mRS 3–5) reported worse HRQoL across multiple domains compared with those with favourable outcome, as shown in table 2. Patients with poor functional outcome also scored worse on assessments of PSCI (median MoCA: 17 vs 24; p<0.01), PSF (median FAS: 21 vs 16; p<0.01) and depression (median PHQ-9: 3 vs 2; p<0.01) (table 2).

**Table 2:** Associations between dependency in ADL and follow-up assessments at 3–4 years with multiple linear regression analysis (n=167)

Barthel Index, total score	B	95% CI for B	SE B	β	R^2^	ΔR^2^
Model					0.68	0.66
Age	−0.082	−0.27 to 0.11	0.10	−0.05		
Sex, male	−3.69	−8.00 to 0.63	2.18	−0.08		
NIHSS at follow-up	−3.81*	−4.77 to −2.85	0.49	−0.53		
CCI at baseline	−2.53†	−4.22 to −0.83	0.86	−0.14		
Living with care at baseline‡	−18.34*	−26.95 to −9.74	4.35	−0.21		
Living alone at follow-up‡	0.34	−4.23 to 4.91	2.31	0.01		
Recurrent stroke‡	−8.15^§^	−14.92 to −1.39	3.31	−0.12		
FAS, total score	−0.65*	−1.05 to −0.26	0.20	−0.25		
MoCA, total score	0.37	−0.13 to 0.88	0.25	0.10		
PHQ-9, total score	0.78	−0.02 to 1.58	0.40	0.14		

Barthel Index: scale 0–100 where 0 is maximum dependency in ADL and 100 is independence

* p<0.005 (corresponding to the Bonferroni-adjusted p value)

† p<0.01

‡ No as reference category

§ p<0.05

ADL, activities in daily living; NIHSS, National Institutes of Health Stroke Scale; CCI, Charlson Comorbidity Index; FAS, Fatigue Assessment Scale (fatigue); MoCA, Montreal Cognitive Assessment (cognition); PHQ-9, Patient Health Questionnaire (depression).

**Table 3:** Ordinal multivariable regression analysis of associations between follow-up assessments and functional outcome (mRS*) at 3–4-year follow-up after stroke (n=167)

	HR (95% CI)	P value
Age at baseline	0.99 (0.97 to 1.02)	0.66
Sex, male	0.61 (0.32 to 1.16)	0.32
NIHSS at follow-up	1.59 (1.33 to 1.89)	<0.001
CCI at baseline	1.31 (1.02 to 1.69)	0.03
Living with home care or at nursing home at baseline†	21.74 (5.08 to 90.90)	<0.001
Living alone at follow-up	1.48 (0.75 to 2.89)	0.26
Recurrent stroke†	3.05 (1.10 to 8.47)	0.03
FAS, total score	1.08 (1.02 to 1.15)	0.009
MoCA, total score, inverse HR	1.09 (1.01 to 1.18)	0.03
PHQ-9, total score	0.96 (0.86 to 1.08)	0.53

Bonferroni-adjusted p value=0.005 for these analyses.

* mRS used as an ordinal variable; scale 0–5 (only survivors included)

† No as reference category

CCI, Charlson Comorbidity Index; FAS, Fatigue Assessment Scale (fatigue); MoCA, Montreal Cognitive Assessment (cognition); mRS, modified Rankin Scale; NIHSS, National Institutes of Health Stroke Scale; PHQ-9, Patient Health Questionnaire (depression).

**Table 4:** Ordinal multivariable regression analysis of associations between follow-up assessments at 3–4 years and HRQoL (SF-36, question 1) (n=167)

	HR (95% CI)	P value
Age at baseline	1.02 (0.99 to 1.05)	0.15
Sex, male	0.69 (0.37 to 1.29)	0.24
NIHSS at follow-up	1.04 (0.90 to 1.20)	0.60
CCI at baseline	1.03 (0.81 to 1.32)	0.79
Living with home care or at nursing home at baseline*	1.75 (0.49 to 6.25)	0.39
Living alone at follow-up	1.17 (0.60 to 2.27)	0.64
Recurrent stroke*	0.78 (0.29 to 2.11)	0.63
FAS, total score	1.15 (1.08 to 1.22)	<0.001
MoCA, total score, inverse HR	1.04 (0.97 to 1.12)	0.25
PHQ-9, total score	1.03 (0.91 to 1.16)	0.65

Bonferroni-adjusted p value=0.005

* No as reference category

CCI, Charlson Comorbidity Index; FAS, Fatigue Assessment Scale (fatigue); HR, hazard ratio; HRQoL, health-related quality of life; MoCA, Montreal Cognitive Assessment (cognition); NIHSS, National Institutes of Health Stroke Scale; PHQ-9, Patient Health Questionnaire (depression); SF-36, Short Form Survey.

Among the 167 stroke survivors who completed all assessments at 3–4-year follow-up, NIHSS at follow-up was independently associated with worse functional outcome (mRS), also after adjustment for above-mentioned covariates (HR: 1.59; 95% CI 1.33 to 1.89; p<0.001). PSF (FAS; HR: 1.08; 95% CI 1.02 to 1.15; p=0.009), recurrent stroke (HR: 3.05; 95% CI 1.10 to 8.47; p=0.03) and cognitive function (MoCA; inverse HR: 1.09; 95% CI 1.01 to 1.18; p=0.03) were also associated with worse functional outcome but did not reach the Bonferroni-corrected alpha level (p=0.006) for these analyses. Meanwhile, dependency in ADL (BI) was significantly associated with NIHSS at follow-up (B: −3.81; 95% CI −4.77 to −2.85); living with care or in a care facility at baseline (B: −18.34; 95% CI −26.95 to −9.73), and PSF at follow-up (B: −0.65; 95% CI −1.05 to −0.26).

At the 3–4-year follow-up after first-ever stroke, 134/195 (69%) of the stroke survivors who had completed the SF-36 question 1 reported good to excellent overall health status. Among those with fair to poor self-reported health (n=61), diabetes mellitus (34% vs 14%; p=0.001) was more common at baseline. At follow-up, those with fair to poor self-reported health status had a higher degree of neurological impairment (median NIHSS: 2 vs 1; p<0.001) as well as higher degrees of PSF (median FAS: 24 vs 15; p<0.001), cognitive impairment (median MoCA: 23 vs 25; p<0.001) and depressive symptoms (median PHQ-9: 6 vs 2; p<0.001).

In the ordinal regression analysis of HRQoL according to SF-36, PSF (according to FAS) at follow-up was alone independently associated with worse HRQoL (HR: 1.15; 95% CI 1.08 to 1.22; p<0.001) after adjusting for baseline variables and other outcome measures.

Regression models and results are presented in tables2^4^.

## Discussion

In this comprehensive, population-based study of three important neuropsychiatric complications after stroke and their relationship to functional outcome and HRQoL, we found that cognitive impairment, PSF, and/or depression are prevalent in at least half of survivors 3–4 years after first-ever stroke. We noted a significant overlap and correlation between PSF and depression as defined by common clinical and research measures and that PSF may be associated with dependency in ADL and worse HRQoL among stroke survivors.

Significant PSF was reported by approximately one in four survivors at follow-up, which is lower than a recently reported prevalence of 52% at 5 years in another Swedish cohort, also using FAS with the same cut-off.^33^ This difference may be in part due to differences in attrition at follow-up (78% response rate in our present study vs 42% response rate).^33^ Somewhat surprisingly, the cohort in our present study was older and had higher stroke severity but yet had a lower PSF prevalence compared with the previous study.^33^ As in prior estimates of PSF, this highlights the variance in prevalence among populations—in this case despite the use of the same measurement and in nearby regions.^6^

As in the aforementioned Swedish study,^33^ we could not identify significant predictors for PSF among traditional vascular risk factors. However, we did not measure body mass index—which has been implicated as a risk factor for PSF,^34^ or specific measurements of obstructive sleep apnoea syndrome.

As mentioned above, PSF and depression were often concomitant in our cohort, which has previously been described.^12^ Among those with significant PSF, depression was present in 43% in our cohort, which is comparable to estimates from the beginning of the millennium^35 36^—corroborating that there also appears to be a subgroup of stroke survivors with significant PSF without depressive symptoms. Nonetheless, 20 of the 21 stroke survivors with depression in our cohort had concomitant significant PSF. Notably, the most reported symptom of depression was feeling tired or having little energy. This illuminates the well-known difficulty in separating the entities of PSF and depression and there is a need for specific definitions and measurements of PSF. While the overlap between PSD and PSF was pronounced, differentiating PSF from PSF with concomitant depression is an important distinction when exploring optimal interventions for PSF.

While PSF has previously been demonstrated to negatively affect functions in complex ADL,^12^ our results also suggest an impact of PSF on basic ADL as measured with the Barthel Index, although the considerable floor and ceiling effects of the Barthel Index measurement^37^ and a possible collider bias situation between depression and PSF must be considered, indicating that further validation is needed.

The prevalence of depression among stroke survivors in our cohort was relatively low compared with other epidemiological studies, at 11% compared with around 27% when pooled in a meta-analysis.^38^ However, most of these previous studies were conducted within 1 year of stroke onset, and longer-term data on PSD are scarce and needed.^38^

Mild or more pronounced cognitive impairment was present among 54% of the followed up participants in our cohort, which is in the higher range of previous estimates for the long term after stroke, particularly considering that cognitive impairments are often described as being transitory in the short term after stroke.^39^ However, several prior long-term studies do not include mild cognitive impairment.^13^ Our detected prevalence is similar to prior 10-year estimates of mild PSCI from our region.^14^ As expected, age and stroke severity were independent predictors of PSCI, but no additional independent predictive value was detected from traditional vascular risk factors, where diabetes mellitus has previously been implicated in other studies.^40^

While survivors with PSCI were older than those without, the age distribution was similar among those with and without both PSF and depression. In prior PSF and PSD studies, age has not been identified as an independent risk factor for the conditions.^41^ This may suggest separate biological or psychosocial mechanisms for PSCI compared with the other neuropsychiatric complications.

Unlike in many other studies,^15^ PSCI was not independently associated with worsened functional outcome, after correction for age, stroke severity and PSF. This may be due to the added control for PSF, but since there was considerable overlap and correlation between PSCI and PSF—this may in part be due to collider bias, and low statistical power may also have influenced our results. In univariate analyses, both PSCI and PSF were significantly associated with worse HRQoL, and dependency in ADL.

Other potential contributing causes of cognitive impairment, fatigue and depression in our cohort include pain and potential post-stroke epilepsy.^42 43^ Post hoc analyses showed that 71 of the 202 stroke survivors (35%) reported either shoulder pain or other pain at follow-up when specifically asked about post-stroke sequelae. Among respondents who reported pain, the prevalences of PSCI, PSF and PSD were 58%, 37% and 19%, respectively. Meanwhile, among the 42/202 (21%) individuals in the total cohort with epilepsy according to chart review, the rates were 78%, 35% and 11%, respectively. However, absolute numbers of respondents with epilepsy were small (18–20 responding stroke survivors to the three assessments).

Strengths of the current study include the following: (1) population-based data; (2) a comprehensive assessment of neuropsychiatric sequela with comparisons between conditions; (3) the use of commonly used measurements for PSCI, PSF and depression; (4) long-term data measuring the chronic phase of stroke and likely thereby excluding potential acute transient fatigue or cognitive impairments.^39 40^ Meanwhile, limitations include the following: (1) a non-negligible proportion of individuals lost to detailed follow-up; (2) the use of FAS for PSF, which is commonly used but may need further comparisons with other measures in upcoming PSF research; (3) the lack of data on pre-stroke function, cognition, fatigue and depression; (4) the lack of a control population for comparison of prevalences of the neuropsychiatric syndromes among age-matched individuals without stroke. However, a prior study from the same study area of a population-based cohort with first-ever stroke in 2001–2002 demonstrated similar rates of cognitive impairment overall among age-matched controls and 10-year stroke survivors, even though there was a higher risk of severe cognitive impairment among stroke survivors.^14^

In summary, our population-based study demonstrates that PSF, PSCI and depression are common long-term complications of first-ever stroke. PSF is associated with worse HRQoL and basic ADL, but there is also a correlation and overlap between PSF and depression that needs to be fully understood. Further research into modifiable causes, interventions and biological mechanisms of PSF is warranted.

## Supplementary material

10.1136/bmjopen-2024-096908 online supplemental file 1

## Data Availability

Data are available upon reasonable request.
