# Supplementary material for: Neuropsychiatric complications 3–4 years after stroke: a population-based study of fatigue, depression and cognition
Source: BMJ Open. 2025 Jul 6;15(7):e096908. doi: 10.1136/bmjopen-2024-096908 (PMC12230960; doi:10.1136/bmjopen-2024-096908)
Supplement: online supplemental file 1 [file bmjopen-15-7-s001.docx]

Supplemental Table 1. Binomial regression of baseline predictors of post-stroke fatigue at 3-4 years

|  | HR (95% CI) | *p* |
| --- | --- | --- |
| Age | 0.99 (0.95-1.02) | 0.45 |
| Sex, male | 1.61 (0.76-3.43) | 0.22 |
| Stroke subtype (ICH as reference) | 0.71 (0.21-2.48) | 0.59 |
| NIHSS | 1.04 (0.98-1.11) | 0.17 |
| CCI | 0.91 (0.63-1.30) | 0.60 |
| Living with home care or at nursing home | 0.58 (0.13-2.52) | 0.46 |
| Recurrent stroke | 1.58 (0.50-4.98) | 0.44 |
| Hypertension | 3.95 (1.35-11.59) | 0.01 |
| Diabetes mellitus | 1.92 (0.74-4.97) | 0.18 |
| Heart disease | 1.02 (0.45-2.29) | 0.97 |
| Hypercholesterolemia | 1.17 (0.54-2.54) | 0.60 |
| Active smoking | 1.42 (0.53-3.78) | 0.48 |

ICH: intracerebral hemorrhage; NIHSS: National Institutes of Health Stroke Scale (stroke severity); CCI: Charlson Comorbidity Index (comorbidities).

All covariates are baseline data other than recurrent stroke which was determined at follow-up.

Supplemental Table 2. Binomial regression of predictors of post-stroke depression at 3-4 years

|  | HR (95% CI) | *p* |
| --- | --- | --- |
| Age | 0.94 (0.90-0.99) | 0.01 |
| Sex, male | 1.14 (0.39-3.37) | 0.81 |
| Stroke subtype (ICH as reference) | 1.30 (0.24-7.14) | 0.76 |
| NIHSS | 1.04 (0.95-1.13) | 0.39 |
| CCI | 1.51 (0.95-2.41) | 0.08 |
| Living with home care or at nursing home* | - | - |
| Recurrent stroke | 2.80 (0.69-11.33) | 0.15 |
| Hypertension | 3.28 (0.74-14.61) | 0.12 |
| Diabetes mellitus | 2.06 (0.59-7.21) | 0.26 |
| Heart disease | 0.70 (0.21-2.36) | 0.56 |
| Hypercholesterolemia | 1.29 (0.42-3.95) | 0.66 |
| Active smoking | 2.14 (0.63-7.34) | 0.22 |

* omitted from analysis due to few total cases in each category

ICH: intracerebral hemorrhage; NIHSS: National Institutes of Health Stroke Scale (stroke severity); CCI: Charlson Comorbidity Index (comorbidities).

All covariates are baseline data other than recurrent stroke which was determined at follow-up.

Supplemental Table 3. Binomial regression of predictors of post-stroke cognitive impairment at 3-4 years

|  | HR (95% CI) | *p* |
| --- | --- | --- |
| Age | 1.08 (1.04-1.12) | <0.001 |
| Sex, male | 1.45 (0.66-3.21) | 0.35 |
| Stroke subtype (ICH as reference) | 0.69 (0.21-2.22) | 0.53 |
| NIHSS | 1.13 (1.04-1.23) | 0.003 |
| CCI | 0.84 (0.60-1.18) | 0.31 |
| Living with home care or at nursing home | 2.84 (0.29-28.30) | 0.37 |
| Recurrent stroke | 5.28 (1.21-23.01) | 0.03 |
| Hypertension | 2.40 (0.98-5.88) | 0.06 |
| Diabetes mellitus | 1.76 (0.60-5.19) | 0.31 |
| Heart disease | 1.13 (0.49-2.63) | 0.78 |
| Hypercholesterolemia | 0.79 (0.36-1.74) | 0.31 |
| Active smoking | 1.91 (0.63-5.81) | 0.26 |

ICH: intracerebral hemorrhage; NIHSS: National Institutes of Health Stroke Scale (stroke severity); CCI: Charlson Comorbidity Index (comorbidities).

All covariates are baseline data other than recurrent stroke which was determined at follow-up.
