# Supplementary material for: Neuropsychiatric complications 3–4 years after stroke: a population-based study of fatigue, depression and cognition
Source: BMJ Open. 2025 Jul 6;15(7):e096908. doi: 10.1136/bmjopen-2024-096908 (PMC12230960; doi:10.1136/bmjopen-2024-096908)
Supplement: Supplementary file 2 [file bmjopen-2024-096908.reviewer_comments.pdf]

PEER REVIEW HISTORY

BMJ Open publishes all reviews undertaken for accepted manuscripts. Reviewers are asked to complete a checklist review form and are provided with free text boxes to elaborate on their assessment. These free text comments are reproduced below.

ARTICLE DETAILS

Title (Provisional)

Neuropsychiatric complications 3-4 years after stroke: A population-based study of fatigue, depression and cognition

Authors

Aked, J; Delavaran, Hossein; Wennerström, Fredrik; Lindgren, Arne

---

VERSION 1 - REVIEW

**Reviewer**	**1**
**Name**	**Creutzfeldt, Claire**
**Affiliation**	**University of Washington Seattle**
**Date**	**05-Mar-2025**
**COI**	**None**

The authors collect interesting outcome measures 3.2 years after stroke. My biggest concern is that the cohort is not described in a way that the reader can judge representativeness, in other words, inclusion criteria are not mentioned and (other than Table 1) the overall baseline characteristics of the cohort are not mentioned. I can see in Table 1 that NIHSS was generally low - mean (I assume this is mean) of 4, so wonder if people unable to report symptoms were excluded? Were surrogates involved? All of this needs to be clarified, I don't think you can ask the reader (or reviewer) to read the referenced previous publications.

**Reviewer**	**2**
**Name**	**Aghoram, Rajeswari**
**Affiliation**	**Jawaharlal Institute of Postgraduate Medical Education and Research, Neurology**
**Date**	**27-Apr-2025**
**COI**	**None**

The authors present medium term outcomes among first ever stroke survivors in the community. I have certain comments and suggestions to make.

1. The original cohort has been described and referenced. However the paper on temporal trends quotes the number of stroke patients identified 2015-2016 as being 456 but in the current paper as well as the other one long term outcomes this has been quoted as 400. Kindly explain the discrepancy.

2. The median study age of the participants is about 72 years. The relevance of the cognitive impairment, fatigue and depression can only be understood of the experiences of a control population from the same context. Kindly discuss this aspect.

3. The 2024 paper on the same cohort shows that 73% had good outcomes on mRS, but the EQ5D shows that pain was a significant problem with a little over 1/2 complaining of pain being a problem. Can the authors share some insight on how this is affecting the fatigue and depression? If there are details about the type of pain are available then that will also be nice to note.

4. A table of the current status of all the participants including the various scores if presented will be a good addition to provide an easier understanding of the subsequent presentations.

5. There has been some reports on association of post stroke epilepsy and neuropsychiatric symptoms (Priya DI et al Neurol Sci. 2021). Was there any such association found in your cohort?

VERSION 1 - AUTHOR RESPONSE

Reviewer 1:
The authors collect interesting outcome measures 3.2 years after stroke.

Response: We are grateful for the reviewer's positive comments.

My biggest concern is that the cohort is not described in a way that the reader can judge representativeness, in other words, inclusion criteria are not mentioned and (other than Table 1) the overall baseline characteristics of the cohort are not mentioned.

Response: We appreciate the reviewer's insight, and that the cohort's representativeness is of utmost importance and must be clear for the reader. We have adjusted the manuscript to include all inclusion and exclusion criteria as follows:

Methods, Population-Based Cohort, 1st paragraph, page 5, lines 8-14:
Briefly, we recruited **adult** individuals **with first-ever stroke according to the WHO definition (17)** between March 1, 2015, and February 29, 2016, in the local catchment area of Skåne University Hospital in Lund, Sweden. Recruitment was both prospective and retrospective from multiple overlapping sources, in agreement with proposed gold standard criteria for epidemiological stroke studies (18). Individuals with ischemic stroke (IS) or intracerebral hemorrhage (ICH) were included, but not those with subarachnoid hemorrhage (SAH). **Traumatic and iatrogenic stroke cases were also excluded.**

We also added reference number 17 (Hatano S. Experience from a multicentre stroke register: a preliminary report. Bull World Health Organ. 1976;54:541-553) to further clarify that inclusion was contingent on a first-ever stroke according to the WHO definition.

Furthermore, Table 1 has been expanded with a column including baseline characteristics of the initial population-based cohort of 400 stroke survivors to facilitate comparison with the surviving respondents to neuropsychiatric assessments at 3-4 year follow-up. We thank the reviewer for recommending this clarification. The Results section has also been amended with a reference to Table 1 as follows:

Results, Included patients and loss to follow-up, 1st paragraph, page 9, lines 4-5:

Four hundred participants with first-ever stroke were included, among whom 151 (38%) died within 4 years before clinical follow-up. **Baseline characteristics of the total cohort are shown in Table 1.**

I can see in Table 1 that NIHSS was generally low - mean (I assume this is mean) of 4, so wonder if people unable to report symptoms were excluded? Were surrogates involved?

We thank the reviewer for identifying that we omitted the measurement of central tendency for NIHSS. The NIHSS provided in Table 1 is a median, with interquartile ranges in parentheses. This has now been rectified in Table 1.

Table 1 now includes the total baseline cohort, wherein the median NIHSS at baseline was 5. This is comparable to other studies, for instance Saver et al in JAMA Neurology (doi:10.1001/jamaneurol.2019.5061) who demonstrated a nationwide median NIHSS of 4 upon study of the distribution of NIHSS scores in administrative claims in ischemic stroke.

We also understand that the NIHSS assessment at baseline needs to be clarified in our manuscript regarding the present study, we have added the following to the Methods section:

Methods, Baseline Characteristics, 1st paragraph, page 5, lines 19-20:
Stroke symptom severity was assessed using the National Institutes of Health Stroke Scale (NIHSS) **by certified research nurses in-hospital, or by review of medical records when necessary (20)**.

All of this needs to be clarified, I don't think you can ask the reader (or reviewer) to read the referenced previous publications.

We agree with the reviewer that these additions facilitate the reading of the present study and are grateful for the recommendations.

Reviewer 2:
The authors present medium term outcomes among first ever stroke survivors in the community. I have certain comments and suggestions to make.

Response: We thank the reviewer for the following suggestions to improve the manuscript.

---

1. The original cohort has been described and referenced. However the paper on temporal trends quotes the number of stroke patients identified 2015-2016 as being 456 but in the current paper as well as the other one long term outcomes this has been quoted as 400. Kindly explain the discrepancy.

Response: We commend the reviewer for thorough examination of the cohort and are thankful for the diligence to ensure our data are correct. However, in the article *Temporal Trends of Stroke Epidemiology in Southern Sweden* from 2018, two different cohorts from two different time-points are included; one from 2001-2002; and one from 2015-2016. There are 456 individuals in the cohort from 2001-2002, which was not followed up in the present study. In the cohort from 2015-2016 (the cohort followed up in the present study), there are 414 individuals. This includes 14 individuals with subarachnoid hemorrhage, who were excluded from further studies of this cohort because the low number of subarachnoid hemorrhage cases leads to insufficient statistical power for this stroke subtype. Therefore, in this study and previous publications on long-term outcome in this cohort – 400 individuals are included.

---

2. The median study age of the participants is about 72 years. The relevance of the cognitive impairment, fatigue and depression can only be understood of the experiences of a control population from the same context. Kindly discuss this aspect.

Response: We agree that comparison with a control population is a valuable adjunct to prevalence studies. Unfortunately, we did not include an age-matched control population in the present study, which is a limitation. This is now further addressed in the Discussion section as follows:

Discussion, 11th paragraph, page 13, lines 5-10:
iv) **the lack of a control population for comparison of prevalences of the neuropsychiatric syndromes among age-matched individuals without stroke. However, a prior study from the same study area of a population-based cohort with first-ever stroke in 2001-2002 demonstrated similar rates of cognitive impairment overall among age-matched controls and 10-year stroke survivors, even though there was a higher risk of severe cognitive impairment among stroke survivors (44).**

---

3. The 2024 paper on the same cohort shows that 73% had good outcomes on mRS, but the EQ5D shows that pain was a significant problem with a little over 1/2 complaining of pain being a problem. Can the authors share some insight on how this is affecting the fatigue and depression? If there are details about the type of pain are available then that will also be nice to note.

and

5. There has been some reports on association of post stroke epilepsy and neuropsychiatric symptoms (Priya DI et al Neurol Sci. 2021). Was there any such association found in your cohort?
We thank the reviewer for these valuable comments on other possible contributing factors to the neuropsychiatric outcomes in the study. Some data is available on both pain and epilepsy from the same follow-up visits, and is summarised in an addition to the Discussion section:

Discussion, 10th paragraph, pages 12-13, lines 30-32 & 1-5:

Other potential contributing causes of cognitive impairment, fatigue, and depression in our cohort include pain and potential post-stroke epilepsy (42, 43). Post-hoc analyses showed that 71 of the 202 stroke survivors (35%) reported either shoulder pain or other pain at follow-up when specifically asked about post-stroke sequelae. Among respondents who reported pain, the prevalences of PSCI, PSF and PSD were 58%, 37% and 19%, respectively. Meanwhile, among the 42/202 (21%) individuals in the total cohort with epilepsy according to chart review, the rates were 78%, 35% and 11%, respectively. However, absolute numbers of respondents with epilepsy were small (18-20 responding stroke survivors to the three assessments).

---

4. A table of the current status of all the participants including the various scores if presented will be a good addition to provide an easier understanding of the subsequent presentations

Response: We thank the reviewer for pursuing further clarity for the readers, and we understand it as this comment to suggests a table including all participants (not just the respondents). This has been addressed in Table 1 in line with the comments from reviewer 1.

VERSION 2 - REVIEW

Reviewer 2

Name Aghoram, Rajeswari

Affiliation Jawaharlal Institute of Postgraduate Medical Education and Research, Neurology

Date 16-Jun-2025

COI

Thank you for addressing my concerns.
